# Futurology of the Situation of Public Hospitals in Iran Until 2032: Futurology of the Situation of Public Hospitals in Iran until 2032

**DOI:** 10.31661/gmj.v14i.3786

**Published:** 2025-11-05

**Authors:** Golsa Danesh, Somayeh Hessam, Shaghayegh Vahdat, Soad Mahfoozpour

**Affiliations:** ^1^ Department of Health Service Administration, ST.C., Islamic Azad University, Tehran, Iran

**Keywords:** Public Hospitals, Futuristic Studies, Hospital Management

## Abstract

**Background:**

Background: The changes in the role of hospitals in the future require planning for the changes in the structure of hospitals. This study aimed to explore the state of public hospitals in Iran by 2032.

**Materials and Methods:**

Materials and Methods: The participants were 20 hospital management and healthcare experts selected using purposive sampling. Structural interaction analysis and MICMAC software were used for data processing.

**Results:**

Results: The findings indicated the growing budgetary constraints in the health sector due to the increasing economic and health burden of non-communicable diseases and emerging diseases caused by environmental changes, the structure of the purchase of drugs, consumables, and medical equipment, the share of the health sector in the national public budget, providing access to capital/loans for the development of hospital activities by the government, the cost-effectiveness ratio of each service, the overcharged tariff set for hospital services, and public health insurance and a shift from employer-based insurance coverage to government-oriented insurance coverage are the key drivers affecting the state of public hospitals in Iran.

**Conclusion:**

Conclusion: The identified factors play a vital role in the state of public hospitals in Iran. This can be useful for policymakers and hospital managers to recognize the future developments of hospitals and healthcare centers.

## Introduction

The changes in the role of hospitals in the future require long-term and medium-term planning for the necessary changes in the structure of hospitals [1]. Examining and choosing different approaches to make changes in hospitals require extensive investigations in this field and finding the best answer for the conditions and characteristics of a country [2]. Hospital management is a complex and multifaceted process that involves planning, organizing, directing, and controlling resources and activities related to providing healthcare services [3]. The main goal of hospital management is to ensure the provision of high-quality services to patients, improve treatment outcomes [4], and increase organizational efficiency and productivity [5]. This task requires the management of human [6], financial, equipment, and information resources in such a way that all divisions of the hospital, including clinical and administrative departments, work harmoniously and effectively [7]. Hospital managers must have good leadership, decision-making, and problem-solving skills to cope effectively with the numerous and varied challenges of this complex environment [8]. In addition, hospital managers must continuously adapt to rapid changes in medical technology, health laws and regulations, and community needs by carrying out innovations, improving quality and patient safety, managing costs, and creating a positive and sustainable organizational culture. Based on patient and staff feedback and analysis of performance data and indicators [9], hospital managers should be able to make effective strategic and operational decisions [10].

Evaluation of hospitals helps policymakers, doctors, and managers control the performance and accuracy and effectiveness of the payment system [11]. It also creates transparency in the affairs and greater responsibility of people in the organization. It will lead to better performance of the organization, especially in the more important sectors from the perspective of stakeholders and the community [12]. These evaluations play a significant role in achieving both internal and external goals of the organization [13].

Furthermore, evaluations give managers a clear perspective on the efficiency and effectiveness of hospitals and can be useful in clarifying the efficiency and success of the organization as well as the utilization of resources [14].

In general, the discovery and evaluation of organizations, in addition to leading to the promotion and accountability of the organization and public trust in the performance of organizations and efficiency and effectiveness, can significantly contribute to planning and developing goals, to prepare the organization to face complex environmental changes [15].

To this end, using a futures research approach, the present study seeks to predict the most likely indicators related to the state of public hospitals in Iran for the next 10 years and provide some implications for healthcare planners. This study employs a structural analysis approach that addresses the state of the system, the way the drivers affect and are affected, and finally the recognition of the key drivers. Thus, the following questions are addressed in this study:

1. What factors will affect the future of public hospitals in Iran by 2032?

2. Are the factors affecting the state of public hospitals in Iran by 2032 stable or unstable?

3. How will the identified factors affect the state of public hospitals in Iran by 2032?

4. What are the key drivers affecting the state of public hospitals in Iran by 2032?

## Materials and Methods

**Table:** Table1. The Participants’ Demographic Characteristics

Variables	Categories	Frequency	Percentage
Education	Master’s degree	8	40
	Ph.D.	12	60
	< 10 years	7	35
Job experience	11-20 year	8	40
	> 20 years	5	25

**Table:** Table2. Factors Affecting the State of Public Hospitals in Iran

Main factors	Categories	
	Tariff structure	C1
	The structure of the financing system	C2
	The structure of the payment system (strategic service purchase by insurance organizations based on quality and price, and public payment for the price of healthcare services)	C3
	The growing budget constraints in the health sector caused by the increasing economic and health burden of non-communicable diseases and emerging diseases caused by environmental changes	C4
	Providing necessary financial resources (from the public budget, donors, etc.) for the production of hospital goods and services	C5
Economic	The structure of purchasing medicines, consumables, and medical equipment	C6
	Economic sanctions	C7
	The growing inflation rate in the health sector	C8
	The growing inflation rate	C9
	Exchange rate change	C10
	The share of the health sector from the national public budget	C11
	Bank interest rate change	C12
	Providing access to capital/loans for the development of hospital activities by the government	C13
Efficiency	Cost-effectiveness ratio of each service	C14
	Bed occupancy ratio	C15
Political	The inappropriateness of the set tariff for hospital goods, equipment, and services	C16
Health super trends	Universal health insurance and a shift from employer-based insurance coverage to government-oriented (tax-based) insurance coverage	C17
	Population aging and the elasticity on the health system	C18
	Suitability of treatment to the patient's needs	C19
	Waiting time in the emergency room (for triage or patient assignment for admission, discharge, or operating room)	C20
	Comprehensiveness of treatment (attention to prevention, treatment, and rehabilitation)	C21
Effectiveness	The amount of information provided to the patient about treatment techniques and outcomes	C22
	The level of executive managers' attention to satisfaction surveys	C23
	Waiting time for patient admission (inpatient ward or operating room)	C24
Legal	Ineffective hospital budgeting system (public budget, linear budget, and ownership of the budget resulting from savings)	C25
	The obligations of hospitals to comply with scientific and local guidelines agreed by the Ministry of Health and insurance organizations	C26
Technological	Advancement of health information technology (home care, telemedicine, distance education, electronic health records, and electronic prescribing)	C27
	The speed of technology changes	C28
Financial	The ratio of personnel payroll costs to total revenue	C29
	The ratio of the cost of medicine and medical consumables to the total costs	C30
	The ratio of total cost to active beds	C31
	Costs incurred per patient day	C32
	The ratio of the cost of medicine and medical consumables to the total private income	C33
	The ratio of total costs to total revenues	C34
	The ratio of total debts to total assets	C35
	Operating income	C36
Sociocultural	Increased public expectations of the healthcare system	C37
	Changes in population growth	C38
	Changing the disease burden pattern toward chronic diseases	C39

This applied and exploratory study adopted a future research approach. It also used a documentary approach and the Delphi method to identify the most important drivers of the future of public hospitals in Iran.

The Delphi method was chosen due to its effectiveness in gathering expert consensus on future-oriented issues, especially when empirical data is limited or unavailable. This method is widely used in healthcare futures studies to identify and prioritize key drivers.

The members of the Delphi team were selected through (judgmental) purposive sampling based on the selection criteria including theoretical knowledge, practical experience, willingness and ability to participate in the study, and accessibility. The number of experts participating in Delphi rounds is usually less than 50 people and generally 15 to 20 people. Accordingly, 20 academic and research experts were selected as the participants in this study in 2023 (Table-1).

The Delphi process consisted of two rounds. In round one, experts received a summary of literature findings and a structured list of 39 potential drivers. Consensus was defined as ≥70% agreement on the relevance and impact of each factor. No items were dropped during the process. MICMAC software was then used to analyze the influence relationships among the final set of factors. Data analysis revealed 39 primary drivers divided into 9 categories (economic, efficiency, political, health super trends, effectiveness, legal, technological, financial, and sociocultural factors) as shown in Table-2.

### Ethical Approval

This article reported the results of a Ph.D. dissertation approved in 2024. The protocol for this study was approved by the ethics committee code IR.IAU.CTB.REC.1402.009 in the Islamic Azad University of South Tehran Branch. This study was conducted with full compliance with all ethical considerations, including maintaining the confidentiality of the identity information of the participants and obtaining their informed consent.

## Results

**Table:** Table3. The Degree of the Direct Effects of the Factors on Each Other

Row	Factors Affecting Hospital Performance	Total row score (level of influencing)	Total column score (level of influence)
1	Tariff structure	62	49
2	The financing system	66	50
3	The structure of the financing system	55	61
4	The structure of the payment system (strategic service purchase by insurance organizations based on quality and price, and public payment for the price of healthcare services)	46	51
5	The growing budget constraints in the health sector caused by the increasing economic and health burden of non-communicable diseases and emerging diseases caused by environmental changes	59	53
6	Providing necessary financial resources (from the public budget, donors, etc.) for the production of hospital goods and services	45	50
7	The structure of purchasing medicines, consumables, and medical equipment	67	21
8	Economic sanctions	58	51
9	The growing inflation rate in the health sector	71	38
10	The growing inflation rate	60	20
11	Exchange rate change	51	63
12	The share of the health sector from the national public budget	42	26
13	Bank interest rate change	49	59
14	Providing access to capital/loans for the development of hospital activities by the government	41	56
15	Cost-effectiveness ratio of each service	54	54
16	Bed occupancy ratio	50	49
17	The high tariff set for hospital goods, equipment, and services	38	49
18	Universal health insurance and a shift from employer-based insurance coverage to government-oriented (tax-based) insurance coverage	56	39
19	Population aging and the pressure on the health system	49	40
20	Suitability of treatment to the patient's needs	35	40
21	Waiting time in the emergency room (for triage or patient assignment for admission, discharge, or operating room)	40	51
22	Comprehensiveness of treatment (attention to prevention, treatment, and rehabilitation)	32	46
23	The amount of information provided to the patient about treatment techniques and outcomes	32	38
24	The level of executive managers' attention to satisfaction surveys	34	45
25	Waiting time for patient admission (inpatient department or operating room)	38	53
26	Ineffective hospital budgeting system (public budget, linear budget, and ownership of the budget resulting from savings)	48	42
27	The obligations of hospitals to comply with scientific and local guidelines agreed by the Ministry of Health and insurance organizations	45	51
28	Advancement of health information technology (home care, telemedicine, distance education, electronic health records, and electronic prescribing)	56	45
29	The speed of technology changes	54	53
30	The ratio of personnel payroll costs to total revenue	42	51
31	The ratio of the cost of medicine and medical consumables to the total costs	41	49
32	The ratio of total cost to active beds	41	60
33	Costs incurred per patient day	42	50
34	The ratio of the cost of medicine and medical consumables to the total private income	42	57
35	The ratio of total costs to total revenues	42	53
36	The ratio of total debts to total assets	57	72
37	Operating income	37	67
38	Increased public expectations of the healthcare system	43	30
39	Changes in population growth	49	30
Total	Changing the disease burden pattern toward chronic diseases	1869	1869

**Table:** Table4. Variables Affecting the Future State of Public Hospitals in Iran

Factors indirectly influenced	Symbol	Factors influencing indirectly	Symbol	Factors directly influenced	Symbol	Factors influencing directly	Symbol	Rank
376	C36	392	C9	385	C36	379	C9	1
354	C37	370	C7	358	C37	358	C7	2
336	C11	355	C2	337	C11	353	C2	3
318	C3	340	C1	326	C3	331	C1	4
316	C32	337	C10	321	C32	321	C10	5
303	C34	318	C5	304	C34	315	C5	6
302	C14	313	C8	299	C13	310	C8	7
299	C13	302	C36	299	C14	304	C36	8
288	C29	294	C3	288	C15	299	C18	9
287	C15	294	C29	283	C5	299	C28	10
287	C5	293	C28	283	C25	294	C3	11
281	C35	287	C18	283	C29	288	C15	12
279	C25	279	C15	283	C35	288	C29	13
276	C21	277	C11	272	C4	272	C11	14
273	C27	268	C16	272	C8	267	C16	15
269	C4	256	C19	272	C21	262	C13	16
269	C30	256	C39	272	C27	262	C19	17
268	C8	255	C13	272	C30	262	C39	18
267	C6	251	C26	267	C2	256	C26	19
266	C2	248	C4	267	C6	246	C4	20
265	C16	241	C6	267	C33	240	C6	21
263	C31	240	C27	262	C1	240	C27	22
263	C17	233	C12	262	C16	230	C38	23
261	C33	228	C38	262	C17	224	C12	24
257	C1	222	C34	262	C31	224	C30	25
254	C22	222	C35	246	C22	224	C33	26
249	C24	221	C31	240	C24	224	C34	27
238	C28	221	C32	240	C28	224	C35	28
225	C26	220	C30	224	C26	219	C14	29
221	C19	220	C33	214	C19	219	C31	30
220	C20	219	C14	214	C20	219	C32	31
217	C38	210	C17	214	C38	214	C21	32
209	C18	207	C21	208	C18	203	C17	33
208	C23	203	C25	203	C9	203	C25	34
198	C9	197	C37	203	C23	197	C37	35
164	C39	180	C20	160	C39	187	C20	36
138	C12	174	C24	139	C12	181	C24	37
109	C7	169	C22	112	C7	171	C22	38
103	C10	167	C23	107	C10	171	C23	39

**Figure-1:**
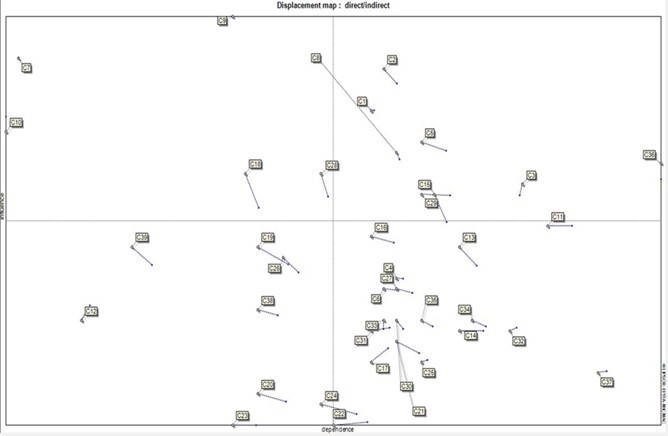
Figure 1. The scree plot for the factors affecting the state of hospitals

**Figure-2:**
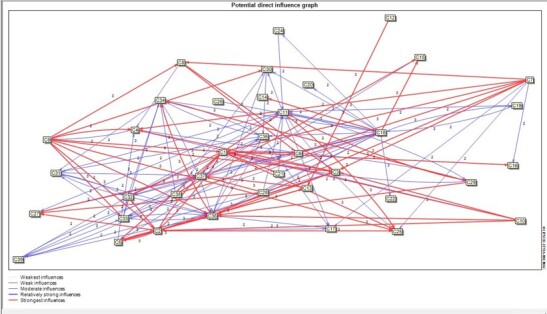
Figure 2. The direct (strongest) influences of the factors

The structural interaction analysis revealed 39 factors underlying the state of public hospitals in Iran by 2032 that were categorized in the form of a 39×39 matrix with 2 iterations and an 89 percent filling degree. The findings also indicated that the extracted variables have some effect on each other and the system is relatively stable. Of a total of 1355 relationships evaluated in the matrix, 166 relationships were zero, indicating that these factors did not affect each other or were not affected by each other. Moreover, 898 relationships with a value of 1 have a weak impact on each other, and 400 relationships with a value of 2 have a relatively strong impact. Besides, 57 relationships have a value of 3, suggesting that the key variables have a great influence on each other. The matrix developed in this study with 2 rotations based on the extracted factors has 100 percent desirability and optimization, which confirmed the acceptable validity of the data collection instruments. Table-3 displays the drivers affecting the state of public hospitals in Iran based on their direct impact. It should be noted that the impact of the drivers that obtained the highest scores may change.

### The Interaction Effects of the Variables

The scatter plot for the distribution of the variables shows the degree of stability or instability of the system. The analysis of the mutual/structural effects of the variables with MICMAC software revealed two types of distribution: stable systems and unstable systems. In the stable system, the variables have an L-shaped distribution. In this system, some variables are affected significantly and some have a great influence. However, unstable systems are more complicated. The variables in this system are scattered around the diagonal axis of the plane, and thus the variables may affect and be affected by each other to a certain degree, which makes it difficult to identify the key variables. A look at the scatter plot of drivers affecting the future of the hospitals shows an unstable system. Most of the variables are scattered around the diagonal axis of the plane. Except for a few variables that have a high impact on the system, the rest of the variables are almost similar to each other in terms of their implications.

Figure-1 shows the distribution pattern of the state of public hospitals in Iran. This scree plot shows an unstable system:

### Classification of Factors Affecting the Perspective of Hospitals

Underlying or influential factors: These factors are more influential but less affected by other factors. Thus, the system depends greatly on these variables. These factors are displayed in the northwest part (second quadrant) of the scree plot. The influential factors are the most critical components because system changes depend on them and the degree of control over these factors is very important. These factors are also considered system input variables. Of the 39 factors addressed in this study, some factors were classified as the drivers influencing the research model including economic sanctions, the increased inflation rate, exchange rate change, population aging, the pressure on the health system, and the speed of technological changes. Two-faceted factors: These factors affect other factors and are affected by other factors at the same time. These factors are placed in the northeast part (first quarter) of the scree plot. These factors are associated with instability because every action and change on them results in a reaction and change in other factors. The factors in the first quarter include the structure of tariffs, the structure of the financing system, the structure of the payment system, the provision of the necessary financial resources (from the public budget, donors, etc.) for the production of equipment and services for hospitals, the increased inflation rate in the health sector, the bed occupancy rate, the ratio of personnel payroll costs to total revenue, and operating income. Two-faceted factors are classified into two categories: risk factors and target factors:

Risk factors: These factors are located above the diagonal line of the northeastern part and have a great capacity to become key players in the system. The structure of tariffs and the structure of the financing system are among the risk factors identified in this study.

Target factors: These factors are placed under the northeast diagonal area of the scree plot (under the diagonal area of the first quadrant). The target factors are the evolutionary outcomes of the system and represent possible goals in a system. The system can be developed by manipulating and making changes in these factors. The target factors identified in this study include the components of the structure of the payment system, the provision of necessary financial resources (from the public budget of the government, donors, etc.) for the production of goods and services of hospitals, the increased inflation rate in the health sector, the bed occupancy rate, the ratio of personnel payroll costs to total revenue, and operating income.

Affected factors or outcome drivers: These factors are located in the southeast part of the scree plot and the fourth quadrant. They have less impact but are affected significantly. Hence, they are very sensitive to the development of influential and two-faceted drivers, and thus they are considered output factors. The output factors identified in this study are growing budget constraints in the health sector caused by the increasing economic and health burden of non-communicable diseases and emerging diseases caused by environmental changes, the structure of purchasing drugs, consumables, and medical equipment, the share of the health sector from the national public budget, providing access to capital/loans for the development of hospital activities by the government, the cost-effectiveness ratio of each service, high tariffs set for hospital goods, equipment, and services, universal health insurance and a shift from employer-based insurance coverage to government-oriented (tax-based)insurance coverage, comprehensiveness of treatment, the amount of the information provided the patient about the treatment procedures and outcomes, the ineffective budgeting system of hospitals, the advancements of health information technology, the ratio of the cost of medicines and medical supplies to the total costs, the ratio of the total costs to the active beds, the costs incurred per patient day, the ratio of the drug cost and medical consumables to total private income, ratio of total cost to total income, ratio of total labilities to total assets, and the increased community expectation of the healthcare system. Independent or exceptional factors: These factors have less influence and are less affected by other factors. They are located in the southwestern part of the scree plot and seem to have no connection with the system at all as they neither stop the main factors nor cause their development in the system. The independent factors extracted in this study included changes in bank interest rates, suitability of treatment to patient needs, waiting time in the emergency room, the level of executive managers' attention to satisfaction surveys, waiting time for patient admission, the obligations of hospitals to comply with scientific and local guidelines agreed by the Ministry of Health and insurance organizations, population growth changes, and disease burden pattern changes towards chronic diseases. However, independent factors also fall under two categories of drivers:

Discrete factors: These factors are located near the coordinate origin in the scree plot. The development of these variables has nothing to do with the dynamics of the current system and they can be removed from the system. No discrete factor was identified in this study.

Secondary leverages factors: Despite being completely independent, these drivers influence others rather than being influenced by other factors. They are located in the southwest of the scree plot and above the diagonal line and can be used as measuring points and benchmarks. The secondary leverages identified in this study are bank interest rate change, suitability of treatment to the patient's needs, the obligations of hospitals to comply with scientific and local guidelines agreed by the Ministry of Health and insurance organizations, changes in population growth, and changing the disease burden pattern towards chronic diseases.

Regulating factors: These factors are located near the center of gravity of the scree plot. They can act successively as "secondary leverages", "weak targets" and "secondary risk drivers". No regulatory factors were identified in this study.

Figure-2 shows a graphic display of drivers affecting the future of the hospital. The figure shows the direct and indirect influences of the drivers on other drivers of the system.

Four key drivers were selected from the 39 drivers examined in this study. These drivers are the most effective key drivers affecting the future state of public hospitals in Iran as shown in Table-4.

## Discussion

Using a futurstic studies framework and the MICMAC approach, this study investigated the perspective of public hospitals in Iran by 2032. MICMAC analysis revealed four categories of factors with economic sanctions and the increased inflation rate being identified as the most influential. Economic sanctions can directly affect imports of medical equipment, drugs, and health technologies. Currency restrictions and reduced access to international resources expose hospitals to serious challenges in providing modern and up-to-date equipment [16]. International sanctions may also increase the cost of importing raw materials and medical products, which in turn can lead to a decrease in the quality of healthcare services and increased costs for patients. In addition, sanctions can reduce the incentive for foreign investment in the Iranian health sector and hence delay the development and improvement of hospital infrastructure [17].

In contrast with other studies, particularly those from Europe and the United States, which emphasize sustainability and technological transformation such as the use of artificial intelligence in hospitals, our study prioritized economic and structural drivers. This reflects the context-specific challenges of Iranian public hospitals but highlights the need for further research on environmental and technological dimensions of futurology.

Artificial intelligence has great potential to improve medical decision-making in the future, but the successful implementation of such systems in medicine requires other things in addition to paying attention to the principles required for any other information system, including organizational, behavioral, cultural, managerial, economic, educational, and technical factors[18].

High inflation rates can dramatically increase hospital operating costs. Rising prices for medicines, medical equipment, and human resources costs make managing the budget and resources of hospitals a big challenge. High inflation rates can also reduce people's purchasing power, consequently affecting the demand for healthcare services and reducing access to health services for vulnerable groups in the community [19].

Moreover, inflation can negatively affect the motivation and satisfaction of hospital staff, because the increased cost of living makes the level of salaries and wages insufficient, which in turn can cause a decrease in the quality of healthcare services provided [20].

Generally, the economic sanctions and growing inflation rates can both simultaneously and mutually affect each other and create many challenges for the management of Iranian hospitals in the future. Managing these challenges requires careful planning, effective policy-making, and efforts to improve efficiency and productivity in the use of limited available resources.

Exchange rate changes can have major effects on the costs and resource management of hospitals. A rise in exchange rates usually raises the cost of importing medical equipment, drugs, and consumables, which can increase hospital operating costs. Exchange rate changes can also lead to a decrease in the ability of hospitals to provide advanced and necessary equipment, a decrease in the quality of healthcare services, and an increase in treatment costs for patients [21].

Furthermore, exchange rate fluctuations can make the financial planning of hospitals more difficult, and thus hospitals require more complex financial and risk management strategies. Population aging is also one of the biggest challenges for Iran's health system in the future [22].

Older adults are more likely to suffer from chronic diseases and long-term care needs, which creates a financial burden and additional pressure on hospitals and the health system. Hospitals need to adapt to these demographic changes through the development of specialized departments such as long-term care, rehabilitation, and social support services. Moreover, there is a growing need for education and training of medical staff specializing in the care of older adults [23].

The speed of technological changes in the field of healthcare has increased dramatically, and hospitals must be able to keep up with these developments. New technologies such as telemedicine, artificial intelligence, data mining, and robotics can help improve service quality, increase efficiency, and reduce costs [24].

However, the adoption and implementation of these technologies require significant investment. Besides, the training of the medical staff is necessary for the effective use of new technologies. Hospitals should also take serious measures in the field of information security and privacy of patients, because new technologies may create privacy and security risks [25].

## Conclusion

The factors identified in this study play a vital role in the state of public hospitals in Iran in different sectors. These findings can be useful for policymakers and hospital managers to recognize the future developments of hospitals and healthcare centers and not be surprised when facing the future.

## Conflict of Interest

The authors declare no conflicts of interest.
